# The role of cancer cell-released extracellular vesicles: have we become closer to cancer pain treatment?

**DOI:** 10.20517/evcna.2024.89

**Published:** 2024-12-26

**Authors:** Iryna A. Khasabova, Sergey G. Khasabov, Donald A. Simone

**Affiliations:** Department of Diagnostic and Biological Sciences, University of Minnesota, Minneapolis, MN 55455, USA.

**Keywords:** Extracellular vesicles, cancer pain, hyperalgesia, TRPV1, head and neck cancer, mouse

## Abstract

The effective management of cancer pain continues to be a challenge because of our limited understanding of cancer pain mechanisms and, in particular, how cancer cells interact with neurons to produce pain. In a study published in *Pain*, Inyang *et al.* used a mouse model of human papillomavirus (HPV1)-induced oropharyngeal squamous cell carcinoma to show a role for cancer cell-derived extracellular vesicles (cancer sEVs) in cancer pain. They found that inhibiting the release of sEVs reduced spontaneous and evoked pain behaviors, and that pain produced by sEVs is due to activation of TRPV1 channels. An innovative approach was the use of publicly available human RNA-sequencing data from unstimulated cultured human dorsal root ganglia (DRG) that were exposed to human head and neck squamous cell carcinoma (HNSCC)-derived sEVs to identify signaling pathways involved in the nascent translation associated with nociception. These studies further our understanding of functional interactions between cancer cells and neurons, and suggest an approach to identify novel targets for the treatment of cancer pain.

## MAIN TEXT

It is now well known that small extracellular vesicles released from cancer cells (cancer sEVs; size 30-150 nm) carry a wide array of biomolecules, including lipids, proteins, and nucleic acids, that promote tumor cell proliferation, invasion, and metastasis^[1]^, as well as axonogenesis^[2]^. Only recently has the role of cancer sEVs in cancer pain been established^[3-5]^. In their manuscript, Inyang *et al.* masterfully expanded on previously published evidence that cancer sEVs contribute to cancer pain, in particular, pain associated with head and neck squamous cell carcinoma (HNSCC)^[6]^.

Cancer pain is complex and, in addition to nociceptive and neuropathic components, involves unique oncogenic factors. sEVs secreted by cancer cells are one such factor that, by increasing the sensitivity of surrounding nociceptors, leads to pain and hypersensitivity^[5,7]^. Considering that patients with HNSCC usually complain of spontaneous ongoing pain, as well as evoked pain hypersensitivity^[8,9]^, it is crucial that Inyang *et al.* measured both types of pain to prove the pronociceptive effect of cancer sEVs using a murine model of human papillomavirus (HPV1)-induced oropharyngeal squamous cell carcinoma. This increases the translational potential and clinical relevance of the study^[6]^.

Using various complementary approaches, the authors identified and thoroughly tested signaling pathways and clinically relevant targets for cancer sEVs *in vivo*. First, Inyang *et al.* clearly demonstrated that the pronociceptive effect of cancer sEVs is determined by their release from cancer cells^[6]^. Second, the authors showed that cancer sEVs are quickly captured by sensory neurons, inducing the expression of ATF3, a marker of neuron damage. Importantly, they showed that administration of a sEV release inhibitor or implantation of cancer cells that release a limited amount of sEVs (mEERL Rab27a^+/-^ and Rab27b^-/-^ cells) attenuated the development of pain, thus providing convincing evidence for a significant role of cancer sEVs in pain.

Another important and clinically relevant finding of this work is that cancer sEVs produce pain exclusively through open TRPV1 channels, and the role of these channels in the sensitization of primary nociceptors and, consequently, in the occurrence of pain was previously demonstrated in a bone cancer pain model^[7]^. This was elegantly confirmed with QX-314, a positively charged lidocaine derivative that inhibits only neurons with open TRPV1 channels. In addition, TRPV1^-/-^ mice did not exhibit pain behaviors in response to cancer sEVs administration, and the ablation of TRPV1-positive neurons with resiniferatoxin prevented the development of both evoked and spontaneous pain in a murine model of HNSCC. Overall, these findings highlight the important role of TRPV1 channels in cancer-related pain.

A special, unique, and innovative approach of the authors was the use of publicly available human RNA-sequencing data from unstimulated cultured human dorsal root ganglia (DRG), which were exposed to human HNSCC-derived sEVs to identify signaling pathways involved in the nascent translation associated with nociception. These data revealed that eukaryotic initiation factor (eIF) 2 and 4, mammalian target of rapamycin (mTOR), and p70S6 kinase participated in the nascent protein translation associated with nociception. Importantly, the same pathways also trigger the translation of the nascent protein induced by cancer sEVs in mouse DRG neurons *in vitro*. Moreover, these pathways are involved in producing evoked and spontaneous pain behaviors in a murine model of HNSCC. Thus, this extremely important finding has made it possible to identify new clinically relevant targets for managing cancer pain.

Finally, like any outstanding work, the manuscript of Inyang *et al.* opens up new horizons for future research in identifying cancer pain mechanisms and new treatment approaches^[6]^. For example, it is important to identify the difference between sEVs associated with painful and painless cancers such as skin cancer. Another perspective is related to the creation of cancer cell-targeted sEV release inhibitors. For example, is it possible that by blocking the release of EVs that also remove cellular waste and thereby maintain cell viability, cancer cells will self-destruct? This may be a useful and effective adjuvant to chemothrapy.

In summary, identifying pain mediators specific to cancer is an extremely difficult, but very important and necessary task for improving the management of cancer pain and reducing opioid use. Excellent work has been done in the present study, which has allowed us to take a step forward in understanding the mechanisms underlying cancer pain by shedding additional light on the role of cancer sEVs in the progression of evoked and spontaneous pain. Undoubtedly, this work brings us closer to our main goal of safe and effective targeted treatment of cancer pain and the reduction of opioid use.

## DECLARATIONS

### Copyright

© The Author(s) 2024.
